# Comparative Analysis of Additional Measurement Error Introduced by Inductive Current Transformers, Rogowski Coils and Electronic Current Transducer for Harmonics of Distorted Current

**DOI:** 10.3390/s26113546

**Published:** 2026-06-03

**Authors:** Michal Kaczmarek, Michal Ozimek, Jerzy Cal

**Affiliations:** Institute of Mechatronics and Information Systems, Lodz University of Technology, 90-537 Lodz, Polandjerzy.cal@dokt.p.lodz.pl (J.C.)

**Keywords:** inductive current transformer, Rogowski coil, current error, phase displacement, conversion error, phase shift, higher harmonic, current distortion, optional wideband accuracy classes, IEC 61869-1, WB0, WB1, transformation accuracy, conversion accuracy

## Abstract

This paper investigates the accuracy of conventional inductive current transformers (iCTs) and Rogowski coils (RCs) in measuring distorted currents, evaluating compliance with the WB0 (up to the 13th harmonic) and WB1 (up to the 60th harmonic) accuracy classes according to the IEC 61869-1 standard. A custom reference iCT, calibrated via the ampere-turns method to achieve a superior baseline accuracy (0.02%), served as the primary benchmark. A zero-flux electronic transducer was utilized strictly to verify this reference. Despite inherent core nonlinearity, tested conventional iCTs with reduced to minimum secondary burdens successfully met the class 0.5-WB1 requirements. In the case of tested Rogowski coils, the study reveals that their wideband performance depends on physical design of the particular type. High-sensitivity coils suffer from increased parasitic capacitance and self-inductance, causing significant additional phase shift at higher frequencies, whereas low-sensitivity, small-diameter coils offer superior linearity. Overall, the tested RCs generally ensured compliance with the 0.5-WB1 class across the evaluated frequency range, with certain units successfully achieving the more restrictive 0.2-WB1 class. Ultimately, conventional iCTs remain a highly reliable solution for metering purposes in low-voltage networks, while properly selected Rogowski coils provide a valuable alternative for power quality analysis and harmonic distortion measurements.

## 1. Introduction

One of the key factors limiting the transformation accuracy of inductive current transformers (iCTs) is the nonlinearity of the core’s magnetization characteristic. This phenomenon occurs in all ferromagnetic materials and stems from their internal domain structure [1,2]. This phenomenon intensifies with an increase in the secondary winding load and current, when iCT operating point on the magnetization characteristic curve is closer to the saturation (where its shape becomes more nonlinear) [1]. The higher the secondary voltage generated by the iCT, the greater the magnetic flux density in the core, which consequently increases the required magnetic field strength and excitation current. Therefore, an increase of the secondary winding load leads to higher current error and phase displacement for all harmonics already present in the secondary current, while additional higher-order odd harmonics emerge. The accuracy of iCTs is also deeply affected by the frequency of the transformed current. At frequencies lower than the nominal 50 Hz, a higher magnetic flux is required to induce the same secondary voltage, which moves the core’s operating point closer to the saturation region. However, within a certain frequency range extending up to several kilohertz, the transformation accuracy typically improves. This occurs because the required magnetic flux density decreases, which additionally reduces core losses. The increase in magnetizing reactance initially offsets the gradual decline in magnetic permeability, leading to a reduction in the excitation current [1,3]. It is only at much higher frequencies, typically exceeding 10 kHz to 20 kHz depending on the core material, that the accuracy begins to deteriorate again due to the continued drop of the magnetic permeability and the dominance of eddy current losses in the magnetic core. The nonlinear phenomena inherent to the magnetic core must also be evaluated in the context of actual operating environments. In contemporary power systems, the transformation accuracy of iCTs is challenged not only by the presence of highly distorted primary currents caused by the proliferation of nonlinear loads, but also by an array of environmental and operational factors. Consequently, a comprehensive performance assessment requires analyzing the impact of combined influence quantities. This involves evaluating the simultaneous effects of harmonic-rich waveforms, variations in ambient temperature, fluctuations in the fundamental current magnitude, and specific secondary burden conditions. The concurrent presence of these multidimensional factors nonlinearly aggregates transformation errors, significantly compromising the reliability of power quality assessments and requiring rigorous verification protocols [4,5,6]. The impedance characteristics of the secondary circuit play a pivotal role in this error propagation. Specifically, variations in the inductive burden heavily influence the iCTs’ wideband frequency response. An increase in the inductive load exacerbates phase displacement and ratio errors during harmonic current measurements, which presents a substantial barrier to strict grid code compliance [7]. As power electronic converters introduce higher-order spectral components into the network, the focus of measurement accuracy has expanded into the wideband and supraharmonic ranges, extending up to 150 kHz. Operating in this extended frequency spectrum requires specialized measurement systems and reference high-voltage sensing chains to calibrate iCTs according to emerging normative frameworks, such as the IEC 61869 series [8,9]. Addressing these complex measurement challenges necessitates sophisticated laboratory infrastructure and advanced analytical models. Evaluating iCT accuracy under non-sinusoidal conditions requires high-performance, precision harmonic generators capable of synthesizing accurate, wideband test waveforms [10]. Furthermore, novel analytical representation methods are critical for systematically quantifying harmonic measurement accuracy, facilitating a transition from traditional single-frequency indices to comprehensive wideband error mapping [11]. Consequently, to maintain measurement integrity, advanced compensation techniques and refined modeling are indispensable. Implementing digital correction algorithms allows for the dynamic compensation of fundamental and harmonic frequency errors by adjusting for core nonlinearity [12]. Additionally, sophisticated behavioral models of the magnetic core enable the precise estimation and correction of transformation errors, which is a fundamental requirement for maintaining high accuracy in synchronized phasor measurements and line parameter estimation [13].

On the other hand, one of the greatest advantages of Rogowski coils (RCs) is that they lack a ferromagnetic core, inherently avoiding these complex, frequency-depending nonlinearities. They consist of a wire wound in the form of a loop that encloses the primary current-carrying conductor. The magnetic flux is generated directly in the air or in an insulating dielectric material. One of the major issues with RCs when used for current measurements is that its output voltage is proportional to the derivative of the current. Therefore, it is always phase-shifted by +90° relative to the primary current. To obtain an accurate representation of the current waveform, it is necessary to use an integrating circuit (signal conditioner) that compensates for the phase shift and generates a signal (current or voltage, depending on the design) proportional to the current [14]. Another important factor is the RMS value of the coil’s output voltage. A lower value is associated with increased susceptibility of the measurement system to noise and conducted and radiated disturbances, which significantly affect measurement accuracy, particularly at low currents. To mitigate this effect, the coil and output cable are shielded, although the coil’s low sensitivity may prevent us from measuring the current under test. It should be noted that shielding itself introduces a significant parasitic capacitance between the coil winding and the shielding layer, which creates additional resonances in the coil’s frequency response and can reduce the first self-resonant frequency by more than three orders of magnitude, e.g., from ~116 MHz to ~33 kHz as demonstrated by Zhao et al. [15] for PCB-embedded coils. This severely narrows the usable measurement bandwidth and may preclude the use of shielded coils in applications requiring wideband accuracy classes WB3 and above. At higher output voltages of the RC, a greater number of turns is used, which leads to an increase in mutual inductance and, consequently, to an increase in the coil’s sensitivity, defined as a higher output voltage for the same value of *di*/*dt*. At the same time, however, increasing the number of turns causes an increase in self-inductance and parasitic capacitance [16,17]. Each turn forms a capacitance with adjacent turns and layers. The winding capacitance increases linearly with the number of turns, while the series inductance increases with the square of the number of turns [18,19]. In multi-layer coils, resonance occurs between the turns, resulting in distortion of the measurement result. Furthermore, a higher number of turns improves sensitivity when measuring small currents but reduces the coil’s natural frequency. As a result, high-order current harmonics are attenuated or phase-shifted, leading to current to voltage conversion errors. Furthermore, while the absence of a magnetic core eliminates saturation, it introduces specific vulnerabilities related to the geometric configuration and operating environment of the measurement setup. The accuracy of an RC is heavily dependent on the position of the primary conductor within the measuring loop. Off centering the conductor alters the mutual inductance distribution along the winding, leading to localized integration errors. This vulnerability is further compounded when RCs are exposed to combined external stressors, e.g., simultaneous variations in ambient temperature [20]. Their linear response makes them applicable for power system diagnostics, including the continuous monitoring of high-frequency transients, partial discharges, and protective relaying applications [21].

The electronic inductive current transformer (eCT) utilizing zero flux technology facilitates the elimination of nonlinear core magnetization effects by maintaining the magnetic core at a constant operating point near zero flux density through an active feedback loop [22,23,24]. A dedicated detection winding monitors the magnetic state of the core where any deviation caused by the primary conductor results in the appearance of harmonics that are processed by the integrated electronic circuit. This circuit generates a precise compensation current in the secondary winding that produces a magnetic flux to directly oppose the flux from the primary source. Because the core remains within the linear region of the magnetic characteristic the system avoids the generation of parasitic harmonics and maintains superior accuracy across its entire specified dynamic range. The high signal to noise ratio is ensured by the electronic amplification of the compensation signal and its measurement across a precision burden resistor. Unlike conventional iCTs this technology provides stable performance even at high primary current levels because the magnetic core never approaches saturation. The ultimate constraint on the linear operating window is defined by the current sourcing capability of the compensation amplifier. It should also be noted that anisotropic magnetoresistance-based contactless current sensors represent an alternative solution, offering high bandwidth, compact dimensions and high isolation voltage, particularly suitable for medium voltage power electronics application [25].

Previous works by the author M. Kaczmarek provide an analytical framework focused on the transformation accuracy and conversion accuracy of instrument transformers and active transducers under distorted grid conditions. To secure accurate high-frequency signal reproduction, investigations specify the operating properties required for inductive current transformers to achieve transformation accuracy compliant with the IEC 61869-1 WB2 class extension up to 20 kHz, emphasizing the critical role of secondary current self-distortion driven by magnetic core nonlinearity [1]. Because core nonlinearity complicates standard accuracy assessments, a dedicated routine testing procedure was introduced to efficiently evaluate transformation accuracy under harmonic distortion by determining the maximum current error and phase displacement at specific frequencies, thereby bypassing exhaustive full-scale type tests with various phase angles of higher harmonics [3]. Furthermore, the rigorous evaluation of transformation accuracy of iCTs and conversion accuracy of eCTs requires a deep understanding of internal thermal dynamics under actual load conditions [23]. The research established that increase of the internal temperature causes accuracy degradations depending on the technology: in ICTs, the heat significantly narrows the magnetic hysteresis loop, which directly amplifies nonlinear effects and the self-generation of low-order harmonics in the secondary current. Simultaneously, in active electronic transducers, the same internal thermal stress induces significant temperature drifts within the electronic signal conditioning components. Expanding the scope to active sensors, a novel measurement methodology was developed to evaluate the conversion accuracy of integrating circuits dedicated to RCs [14]. Instead of generating problematic high primary currents, this system utilizes an arbitrary waveform generator to supply a proportional reference voltage directly to the voltage-to-voltage and voltage-to-current integrators. This experimental setup allows for precise isolation and measurement of the specific conversion errors and phase shifts of harmonics caused exclusively by the active signal conditioner. Further research evaluated the performance of combined current transducers, specifically Rogowski coils equipped with dedicated signal conditioners [26]. Initial baseline evaluations of the isolated primary sensors confirmed exceptional metrological linearity. An isolated coil with a 22.5 mV/kA sensitivity maintained measured value errors well below ±1% for higher harmonics up to the 50th order and phase shifts not exceeding ±0.2°. Similarly, a second evaluated coil featuring a 100 mV/kA sensitivity sustained conversion errors below ±1% and phase shifts constrained within ±3°. However, the subsequent integration of active signal conditioner demonstrated a significant and hardware-dependent degradation of this innate accuracy. The combined transducer with voltage output exhibited severe performance deterioration. Its measured value errors exceeded ±5% above 500 Hz, and its phase shifts reached up to +360° for high-order harmonics. Conversely, the second combined transducer with current output maintained measured value errors below ±5% and phase shifts within ±8° across the 2.5 kHz bandwidth. These findings indicate that tested analog conditioners severely compromise conversion accuracy of RCs.

## 2. Structure and Principle of Operation of the Tested Current Transducers

Conventional iCTs are among the most used components in power grid measurement and protection systems. Their primary function is to proportionally convert the primary current into a secondary current of lower value (reduced by the current ratio) appropriate for measurement and protection equipment. This enables safe, accurate, and reliable monitoring of the power grid [1,3,4,8]. An iCT operates under conditions similar to a short-circuit on the secondary winding. It consists of:

- A magnetic core, usually toroidal in shape, made of materials with high magnetic permeability, e.g., special electrical steel, permalloy or superpermalloy, a nanocrystalline alloy, or amorphous powder;
- A primary winding, which may be a single conductor, busbar, or several turns of wire;
- A secondary winding, made of copper wire with multiple turns evenly distributed over the surface of the magnetic core.

**Figure 1:**
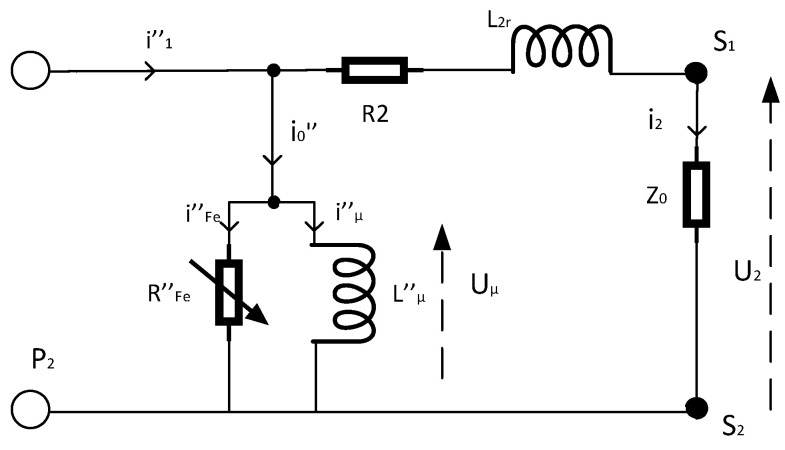
The equivalent circuit of iCT.

The equivalent circuit of iCT is shown in Figure 1.

In Figure 1 the following abbreviations are used (symbols with ″ represent quantities converted to the secondary side of iCT):

$P_{1/2}$—primary winding terminals, $S_{1/2}$—secondary winding terminals;

$i_{0}^{\prime \prime }$—instantaneous value of the magnetic core excitation current;

$i_{1}^{\prime \prime }$—instantaneous value of the primary current;

$i_{2}$—instantaneous value of the secondary current;

$i_{Fe}^{\prime \prime }$—instantaneous value of the current associated with the active power losses in the magnetic core;

$i_{\mu }^{\prime \prime }$—instantaneous value of the magnetic core magnetizing current;

$u_{2}$—instantaneous value of the secondary voltage;

$R_{Fe}^{\prime \prime }$—equivalent resistance of the magnetic core;

$L_{\mu }^{\prime \prime }$—mutual inductance of the iCT’s windings;

$L_{2r}$—leakage inductance of the secondary winding;

$R_{2}$—resistance of the secondary winding;

$u_{\mu }$—instantaneous value of the voltage on the equivalent mutual inductance;

$Z_{0}$—impedance of the secondary winding load—in this case R_0_ resistance is used.

The principle of operation of iCT is based on two fundamental laws of electromagnetism: Faraday’s law of electromagnetic induction and Ampère’s law:

(1) $$\varepsilon =-\frac{d\Phi }{dt}$$

(2) $$\oint H\cdot dl=I_{enc}$$

where ε is the induced electromotive force, and Φ is the magnetic flux linking the winding. H is the magnetic field intensity in the core, and $I_{enc}$ is the total conduction current.

Consequently, the alternating current in the primary winding induces a time-varying magnetic flux within the core. This alternating flux links with the secondary winding, thereby inducing an electromotive force that drives the secondary current. The system operates under the constraint of magnetomotive force (MMF) balance, commonly referred to as ampere-turn equilibrium. Under these conditions, the secondary winding generates a counter-flux that largely opposes and cancels the primary flux, leaving only a residual magnetizing flux necessary for core excitation. Referring to the equivalent circuit model (Figure 1), the secondary current can be analytically derived using the following equation:

(3) $$i_{2}^{\prime \prime }=i_{1}^{\prime \prime }-i_{0}^{\prime \prime }$$

The core excitation current, necessary for establishing the magnetic flux, constitutes the primary source of transformation errors in iCTs. Because the core’s magnetization characteristic is inherently nonlinear, the excitation current is non-sinusoidal. Consequently, even under purely sinusoidal primary current conditions, the resulting secondary current exhibits distortion. Within the quasilinear range of the magnetization curve, this distortion is minimal, though specific harmonic components, such as a measurable RMS values of the 3rd and 5th harmonics, are still present [1,3].

Conventional iCTs are designed to transform sinusoidal current of power system frequency with a given accuracy class. The IEC 61869-2 standard distinguishes a number of accuracy classes, e.g., 0.1, 0.2, 0.5, that differ in permissible limits of current error and phase displacement and evaluated current range, e.g., 0.2 and 0.2 s or 0.5 and 0.5 s.

In the standard IEC 61869-2, current error is defined by the following equation [27]:

(4) $$\Delta I=\;\frac{{k\cdot I}_{2}-I_{1}}{I_{1}}\;\times \;100\%\;$$

where $I_{2}$ is the RMS value of the secondary current, $I_{1}$ is the RMS value of the primary current, and k is the rated current ratio of iCT.

Phase displacement is defined by the following equation [27]:

(5) $$\Delta \phi =\varphi_{2}-\varphi_{1}$$

where $\varphi_{2}$ is the phase angle of the secondary current relative to the reference current, and $\varphi_{1}$ is the phase angle of the primary current relative to the reference current.

The IEC 61869-1:2023 standard introduces additional accuracy classes with an extended frequency range starting from 100 Hz [28]:

- WB0: up to and including the 13th harmonic of the 50 Hz nominal frequency, with maximum current error and phase displacement of ±5%/±5° (to 200 Hz), ±10%/±10° (250–300 Hz), and ±20%/±20° (350–650 Hz);
- WB1: up to and including 3 kHz;
- WB2: up to and including 20 kHz;
- WB3: up to and including 150 kHz;
- WB4: up to and including 500 kHz.

The wideband accuracy classes from WB0 to WB4 are correlated with the basic accuracy classes of iCT determined for standard power frequencies (50 Hz or 60 Hz), for example:

- 0.1-WB1: maximum current error/phase displacement of ±1%/±1° (≤1 kHz), ±2%/±2° (≤1.5 kHz), and ±5%/±5° (≤3 kHz);
- 0.2-WB1: maximum current error/phase displacement of ±2%/±2° (≤1 kHz), ±4%/±4° (≤1.5 kHz), and ±5%/±5° (≤3 kHz);
- 0.5-WB1: maximum current error/phase displacement of ±5%/±5° (≤1 kHz), ±10%/±10° (≤1.5 kHz), and ±10%/±20° (≤3 kHz).

**Figure 2:**
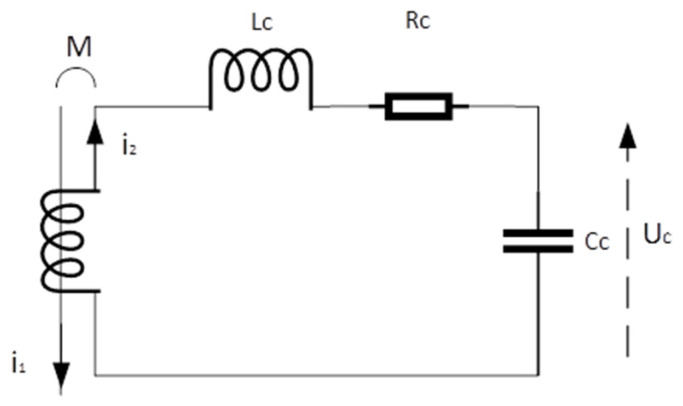
Equivalent circuit of the Rogowski coil.

The Rogowski coil (RC) is an increasingly common AC current sensor, distinguished by its highly linear response and a wide conversion range. This advantage results from the absence of a magnetic core, which eliminates the saturation phenomenon typical of iCTs. The principle was formally detailed by the German physicists Walter Rogowski and Wilhelm Steinhaus in 1912. The RC essentially consists of a helical winding made of thin copper wire wound evenly around a flexible, non-magnetic core, with the return wire routed back through the center of the winding to neutralize interference from external magnetic fields. Figure 2 shows a simplified equivalent circuit.

In Figure 2 the following abbreviations are used:

$i_{1}$—instantaneous value of the primary current;

$i_{2}$—instantaneous value of the secondary current;

M—mutual inductance between secondary winding of RC and the primary conductor;

Lc—self-inductance, leakage inductance of the secondary winding;

$R_{c}$—resistance of the secondary winding;

$C_{c}$—internal parasitic capacitance;

$u_{c}$—output voltage induced in the RC winding.

The operating principle of the RC, similar to that of the iCT, is based on Faraday’s law of induction. An alternating current flowing through a conductor enclosed by the coil generates a time-varying magnetic field that links with the turns of the winding. Consequently, a voltage proportional to the time derivative of the current is induced within the coil. Mathematically, this can be expressed by the equation

(6) $$u_{c}=-M\times \frac{{di}_{1}}{dt}$$

The voltage induced across the terminals of an RC is strictly proportional to the time derivative of the primary current. Therefore, an integration stage is mandatory to reconstruct the original waveform and obtain the true RMS value of higher harmonics. Without it the amplitude of the induced voltage for any harmonic is amplified by a factor proportional to its respective order, owing to the derivative nature of the sensor. Furthermore, as the equivalent circuit incorporates the winding’s self-inductance and inter-turn stray capacitance, the sensor inherently behaves as a distributed-parameter RLC circuit. Consequently, these parasitic elements introduce amplitude errors and phase deviations that progressively compromise the strict linearity of the sensor’s frequency response.

## 3. Methodology and Measurement System Configuration

**Figure 3:**
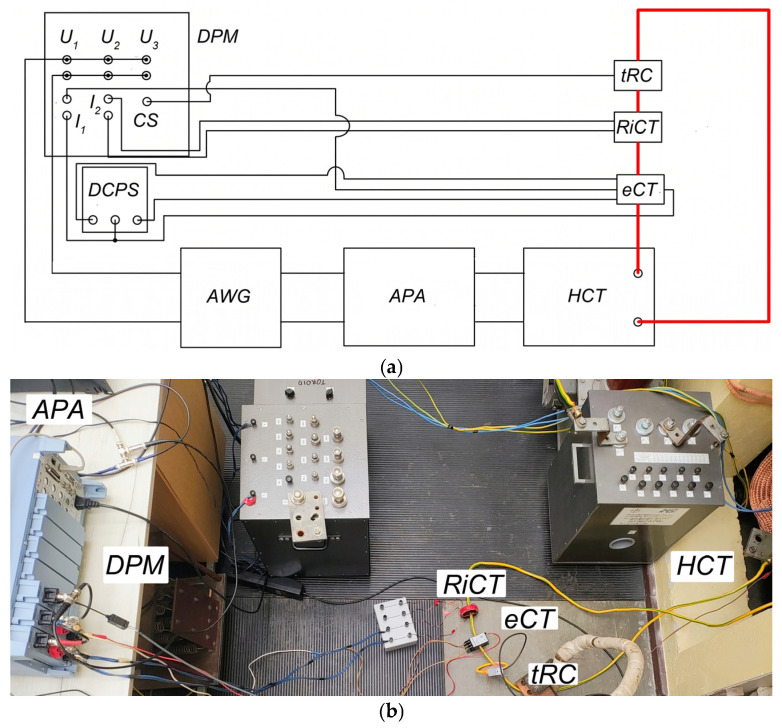
The measurement setup in configuration with Rogowski coil (tRC), reference inductive current transformer (RiCT) and electronic current transformer (eCT): (**a**) connection diagram, (**b**) photo.

Figure 3 shows a simplified connection diagram (a) and photo (b) of used measuring setup.

In Figure 3 the following abbreviations are used: DPM—3 module digital power meter, U—voltage measuring channel of DPM, I—current measuring channel of DPM, CS—current-sense channel of DPM (voltage measuring channel for connection of external current sensor), DCPS—DC power supply for eCT, AWG—arbitrary waveform generator with 2 channels, APA—audio power amplifier, HCT—high-current transformer.

The measurement setup utilized a circuit operating at high currents where the devices under test were connected in series for current evaluation by the DPM. The system was fed by an audio amplifier driven by an arbitrary waveform generator. The DPM measured the RMS values of the current harmonics and their phase angles relative to the reference voltage from the GPA. The current and voltage output signals (current or voltage) from the tested transducers and the RiCT were routed directly to the DPM, a Yokogawa WT5000, for processing and comparison. Regarding its accuracy specifications for harmonic measurements, two key parameters must be distinguished. The base accuracy of the analog input modules in the 1 to 10 kHz band is ±(0.1% of reading + 0.05% of range). The accuracy of the harmonic measurement function inherently decreases in the upper frequency band. This error reaches a maximum of ±(1% of reading + 0.05% of range) for frequencies below 10 kHz. Each module contains a voltage measurement input (U) and a current measurement section. This current section can operate in a direct current measurement mode (I) or accept a voltage signal from an external current sensor (CS). These two current input modes are mutually exclusive within a single module. For data acquisition, the DPM was interfaced with a computer to transmit measurement results in real time directly to an Excel spreadsheet. The acquired data were subsequently formatted and analyzed. Depending on the specific configuration, various transducers were connected across the two available DPM channels (from 3 modules one is used for RiCT). During testing, individual harmonic levels were imposed as specific percentages of the fundamental component. Their assigned values are summarized in Table 1.

In addition, the phase angle of each harmonic with respect to the fundamental component was set to 0°.

Tested current transducers and their technical specification:

- The inductive current transformer (RiCT) used as a reference was developed at the Institute of Mechatronics and Information Systems of Lodz University of Technology, Poland. Rated current ratio: 250 A/5 A. Its wideband transformation accuracy for sinusoidal currents and for harmonics of distorted currents was verified over the range from 50 Hz to 20 kHz using the ampere-turns method, as described in [1]. It meets the requirements of the wideband optional accuracy class 0.1-WB2 of IEC 61869-1 [28], while the current and phase-displacement errors do not exceed ±0.02% and ±0.02° respectively over the indicated frequency range.
- The electronic current transformer (eCT) is a zero-flux device based on fluxgate technology. Its current ratio is 1:1000, with a rated RMS primary current of 200 A and a rated secondary current of 200 mA.
- Three conventional iCTs, all complying with the IEC 61869-2 standard: one with a rated current ratio of 250 A/1 A (TiCT_1) and an accuracy class of 0.5; another 400 A/1 A (TiCT_2) and an accuracy class of 0.5S; and a third with a rated current ratio of 100 A/1 A (TiCT_3) and an accuracy class of 0.2.
- Five Rogowski coils (RCs): the first two (RCd95_1 and RCd95_2) are of the same type, each having a diameter of 95 mm, a current-to-voltage conversion factor of 100 mV/1 kA, and a manufacturer-declared accuracy class of 0.2 according to IEC 61869-10 [29] (which shares the same accuracy requirements as IEC 61869-2 [27]); the third (RCd140_1) has a diameter of 140 mm, a current-to-voltage conversion factor of 100 mV/1 kA, and a manufacturer-declared accuracy class of 0.2 according to IEC 61869-10; the fourth (RCd140_2) has a diameter of 140 mm, a current-to-voltage conversion factor of 500 mV/1 kA, and a manufacturer-declared accuracy class of 0.5 according to IEC 61869-10; and the fifth (RCd70) has a diameter of 70 mm, a current-to-voltage conversion factor of 22.5 mV/1 kA, and a manufacturer-declared accuracy class of 0.5 according to IEC 61869-2.

Table 2 presents the list of abbreviations for the transducers whose measurement results are reported in this paper, along with their main technical specifications derived from the product datasheets.

The testing phase involved an examination of various measurement system configurations encompassing all transducers. The acquired data facilitated a precise analysis of transformation errors inherent to each device type, specifically, current error and phase displacement for the iCTs, alongside current-to-voltage conversion error and additional phase shift for the RCs. Consequently, this yielded a comprehensive performance evaluation of the tested equipment. Furthermore, all experimental measurements strictly adhered to the requirements specified in the IEC 61000-4-7 standard [30]. Three RMS values of the primary current were used in the measurements: 50 A, 100 A, and 150 A.

The RiCT, whose transformation accuracy was established via the rated ampere-turns method, was subsequently verified against the eCT. The current error was calculated utilizing the following equation:

(7) $$\Delta W_{I}\left[\%\right]=\frac{{(I}_{hel}\;\times \;k_{el})-(I_{hw}\;\times \;k_{w})}{\left(I_{hw}\;\times \;k_{w}\right)}\;\times \;100\%$$

where

I_hel_—RMS value of a given harmonic measured by DPM in the secondary current of eCT,

k_el_—current ratio of eCT (1:1000), k_w_—ratio of RiCT,

I_hw_—RMS value of a given harmonic measured by DPM in the secondary current of RiCT.

The following equation was used to calculate the phase-displacement:

(8) $$\Delta \phi_{h}=\varphi_{hel}-\varphi_{hw}$$

where

φ_hw_—phase angle of a given harmonic in the secondary current of the RiCT with respect to the harmonic of the reference voltage at the same frequency,

φ_hel_—phase angle of a given harmonic in the secondary current of the eCT with respect to the same harmonic of the reference voltage.

**Figure 4:**
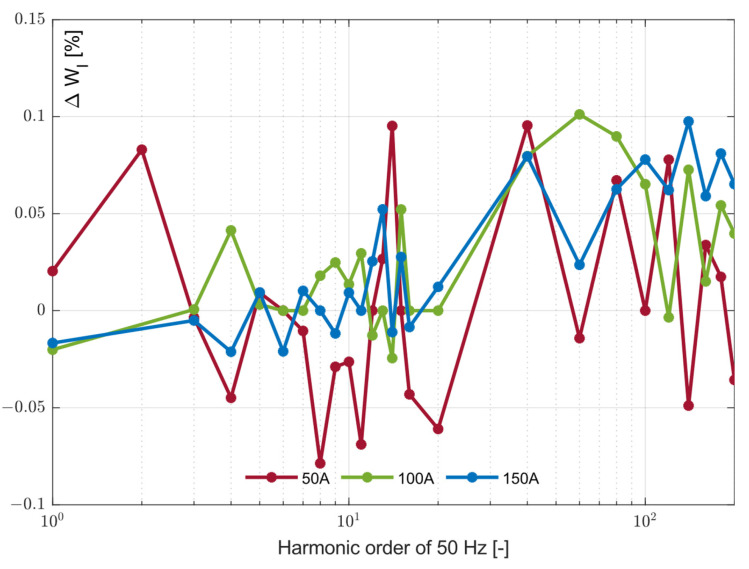
Current error of the eCT relative to the RiCT for primary currents of 50 A, 100 A, 150 A.

**Figure 5:**
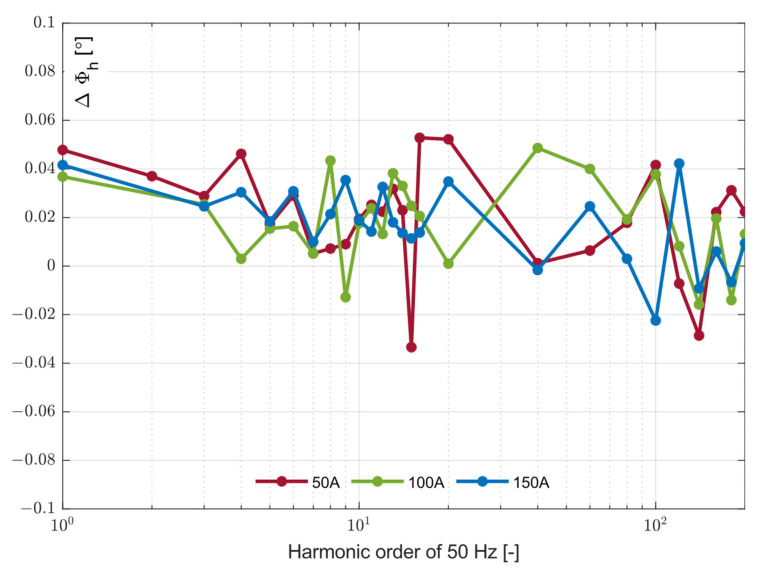
Phase displacement of the eCT relative to the RiCT for primary currents of 50 A, 100 A, 150 A.

Figure 4 and Figure 5 presents the results of the transformation accuracy verification of the RiCT, showing the current error (Figure 5) and phase displacement (Figure 5) between the eCT and the RiCT for primary currents of 50 A, 100 A, and 150 A.

The declared accuracies of both instruments are foundational to the analysis of the eCT’s current error and phase displacement relative to the RiCT ±0.02% and ±0.02° for current error and phase displacement, respectively. The eCT has a declared by manufacturer accuracy of ±0.1%/° up to 5 kHz, and ±2.0%/0.5° within the 5 kHz–100 kHz range. Consequently, the measured deviation between the RiCT and the eCT should nominally remain within ±0.12%/° for frequencies up to 5 kHz and 2.0%/0.5° within the 5 kHz–10 kHz range (form 100th to 200th harmonic order of 50 Hz). Based on these specifications, the observed characteristics fall within the anticipated tolerance range. The observed variations in the current error values (and, to a lesser degree, phase displacement) of the eCT in relation to the RiCT stem from noise and conducted disturbances affecting the DPM measurement channels. This effect is particularly significant when calculating small differences between the compared currents.

The current-to-voltage conversion error of the RC was determined from equation

(9) $$\Delta W_{I}\left[\%\right]=\frac{\frac{U_{hRC}}{k_{RC}\cdot n_{h}}-(I_{h}\cdot k)}{\left(I_{h}\cdot k\right)}\times 100\%$$

where

U_hRC_—RMS value of a given harmonic of the voltage at the output of RC,

k_RC_—rated 50 Hz current-to-voltage conversion factor of tested RC, e.g., 100 mV/1 kA,

I_h_—RMS value of a given harmonic of the secondary current of the RiCT or eCT,

k—current ratio of the RiCT or eCT,

n_h_—order of a given distorted current harmonic.

The following equation was used to calculate the additional phase shift of a given output-voltage harmonic with respect to the input current of the RC:

(10) $$\Delta \phi_{h}={90^{o}-(\varphi }_{hRC}-\varphi_{h})$$

where

φ_hRC_—phase angle of a given harmonic in the secondary voltage of the tested RC with respect to the same harmonic of the reference voltage,

φ_h_—phase angle of a given harmonic in the secondary current of RiCT or eCT with respect to the same harmonic of the reference voltage.

**Figure 6:**
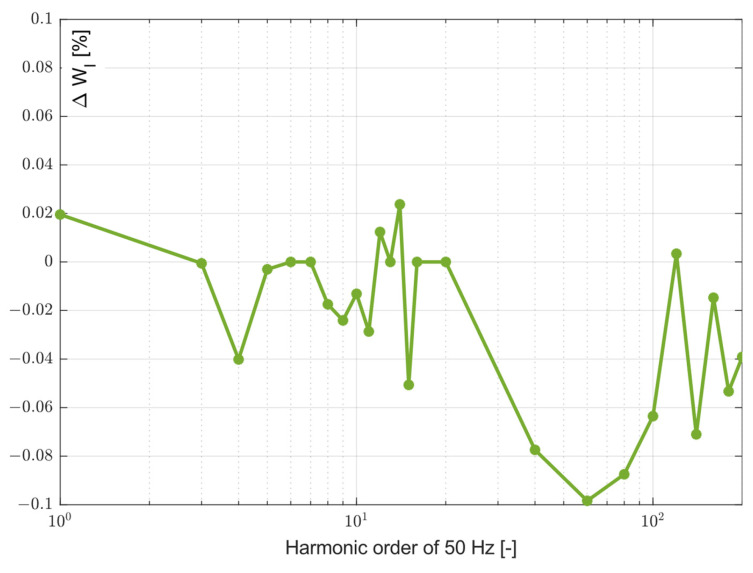
Difference between determined values of the current-to-voltage conversion error of the RCd95_1 relative to eCT and RiCT for a primary current 100 A.

**Figure 7:**
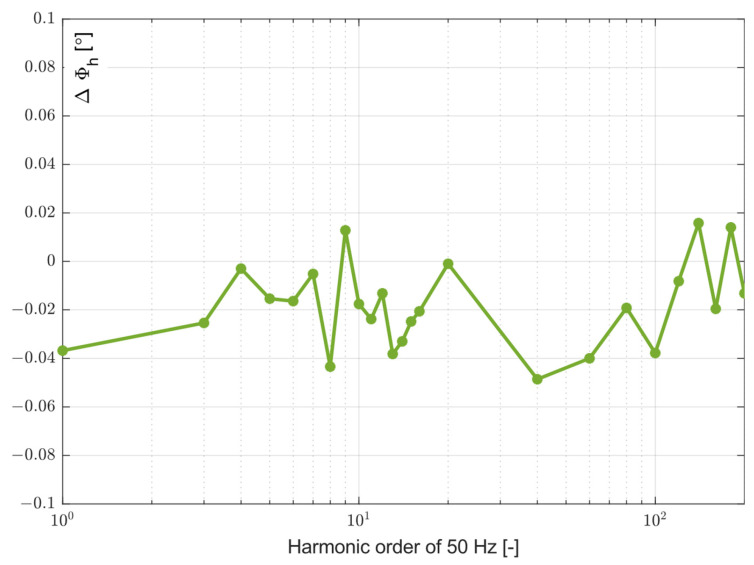
Difference between determined values of the additional phase shift introduced by the RCd95_1 relative to eCT and RiCT for a primary current 100 A.

Verification of the accuracy of the RiCT against the eCT was also based on the results obtained for the same coil, as presented in Figure 6 and Figure 7.

The discrepancies in the current-to-voltage conversion error and the additional phase shift introduced by the tested RC, determined relative to the two reference transformers, do not exceed ±0.1% and ±0.05°, respectively. As previously mentioned, these variations arise from independent conductive disturbances and external electromagnetic noise affecting each DPM measurement channel. These results confirm that the RiCT is applicable as a reference current source for the wideband characterization of the RCs under test.

Based on all results presented in Figure 4, Figure 5, Figure 6 and Figure 7, it can be concluded that the expanded uncertainty of the current error and current-to-voltage conversion error determination does not exceed U = ±0.1%, and the expanded uncertainty of the phase displacement and additional phase shift determination does not exceed U = ±0.05° across the entire tested frequency range up to 3 kHz, at a coverage factor of k = 2.

## 4. Wideband Performance Comparison of Tested Transducers

**Figure 8:**
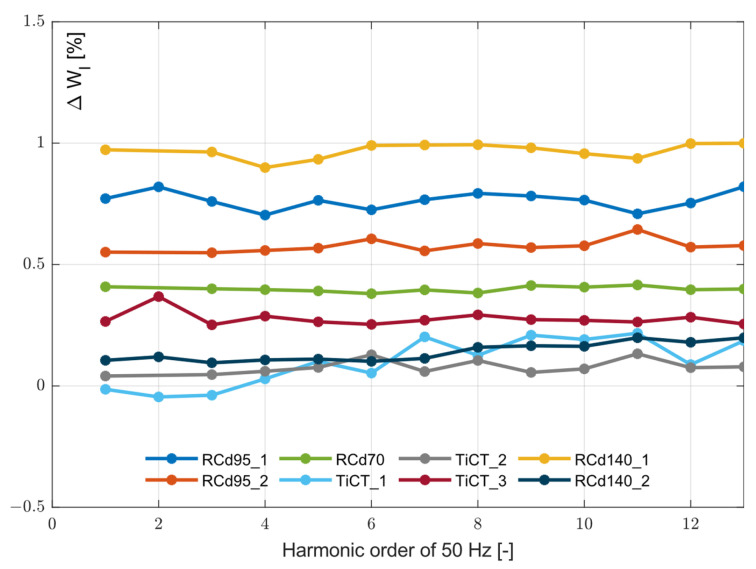
Current-to-voltage conversion (RCs) and current (iCTs) errors for the 1st–13th harmonics, relative to the RiCT at 50 A (WB0 compliance evaluation).

**Figure 9:**
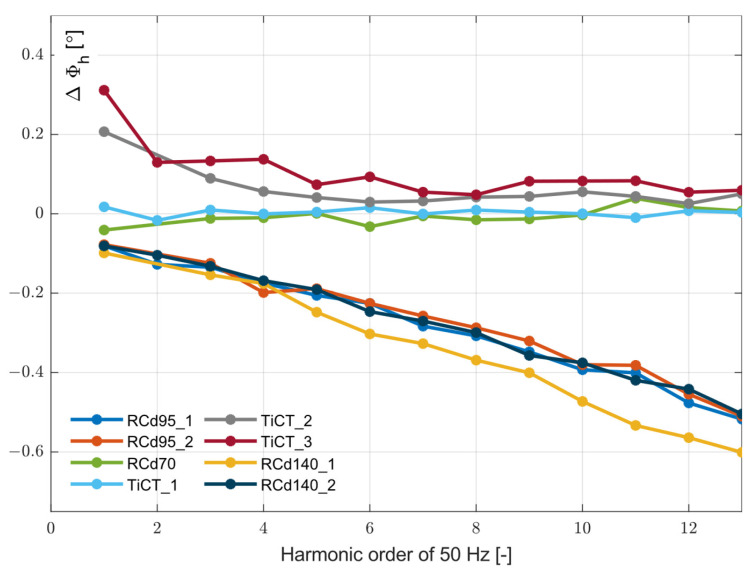
Additional phase shift (RCs) and phase displacement (iCTs) for the 1st–13th harmonics, relative to the RiCT at 50 A (WB0 compliance evaluation).

**Figure 10:**
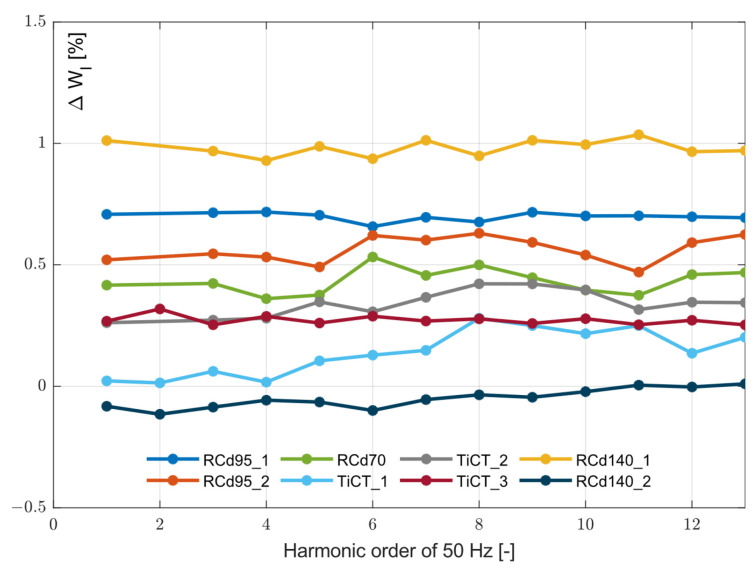
Current-to-voltage conversion (RCs) and current (iCTs) errors for the 1st–13th harmonics, relative to the RiCT at 100 A (WB0 compliance evaluation).

**Figure 11:**
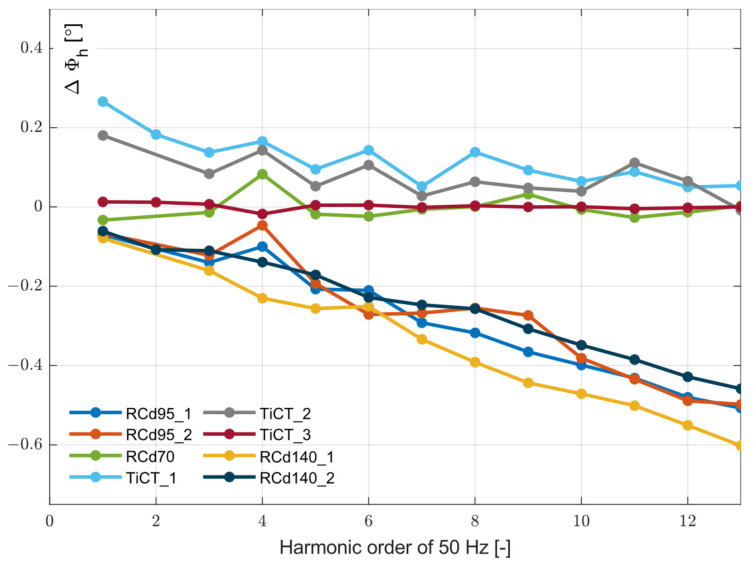
Additional phase shift (RCs) and phase displacement (iCTs) for the 1st–13th harmonics, relative to the RiCT at 100 A (WB0 compliance evaluation).

**Figure 12:**
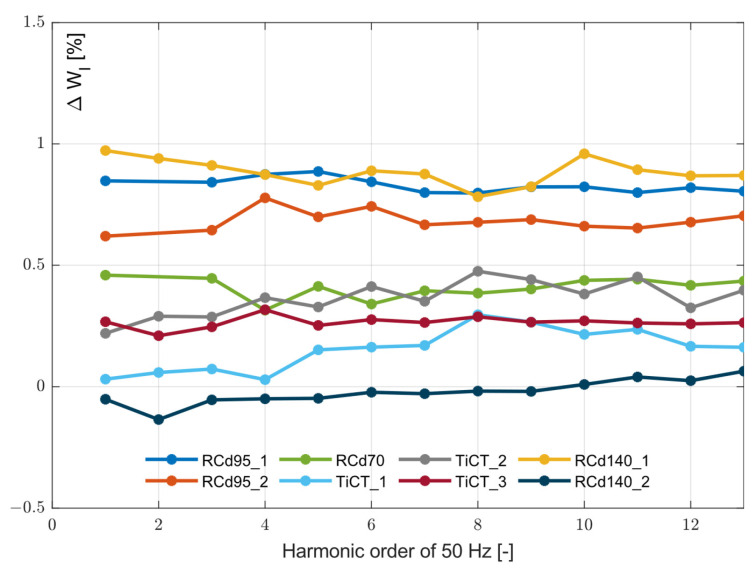
Current-to-voltage conversion (RCs) and current (iCTs) errors for the 1st–13th harmonics, relative to the RiCT at 150 A (WB0 compliance evaluation).

**Figure 13:**
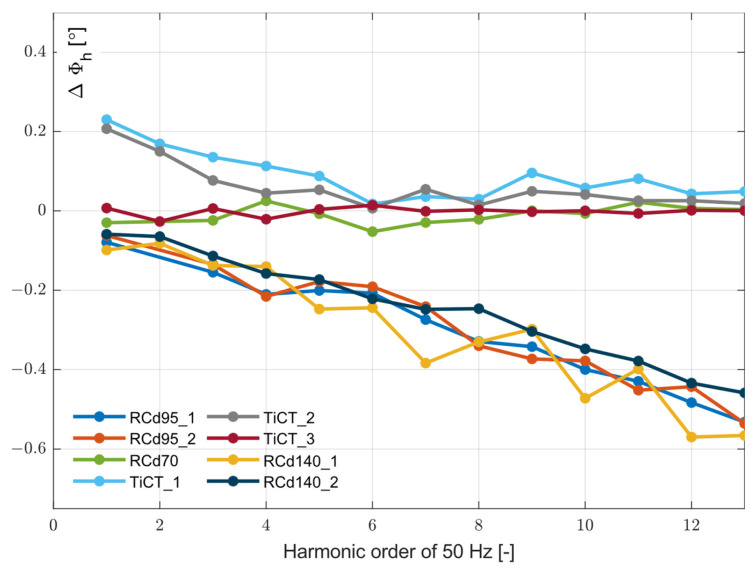
Additional phase shift (RCs) and phase displacement (iCTs) for the 1st–13th harmonics, relative to the RiCT at 150 A (WB0 compliance evaluation).

The wideband characteristics of the tested RCs and iCTs for the 1st–13th harmonics are illustrated in Figure 8, Figure 9, Figure 10, Figure 11, Figure 12 and Figure 13. These figures compare current-to-voltage conversion errors and current errors as well as additional phase shift introduced by tested RCs and phase displacement of iCTs determined relative to the RiCT at three primary current levels: 50 A, 100 A, and 150 A. The results are further analyzed for compliance with requirements of the IEC 61869-1 WB0 accuracy class.

Since WB0 compliance is evaluated for iCTs, their performance must be verified at different current levels, as they are nonlinear devices. Therefore, the final decision requires at least the set of results presented in Figure 8, Figure 9, Figure 10, Figure 11, Figure 12 and Figure 13 for final confirmation and simultaneous verification of both errors in the case of iCTs and RCs. In general, based on the first set of results, it can be seen that iCT current errors are below 0.5%, and RC errors are below 1% in the case of current-to-voltage conversion. However, RCd140_2 shows similar performance to iCTs in that regard, while its additional phase shift increases to −0.5° (as is generally the case for most tested RCs) at the 13th harmonic, whereas the tested iCTs exhibit a linear response below 0.1° over the same range. In this respect, RCd70 performs most comparably to iCTs.

Taking into consideration the results presented in Figure 10 concerning current-to-voltage conversion (RCs) and current (iCTs) errors for the 1st–13th harmonics at 100 A, in comparison to those obtained at 50 A, only the results for TiCT_2 have changed significantly—as the current error values were close to 0% at 50 A, they have now increased to approximately 0.3%. This is due to the fact that in the case of the 400 A/1 A iCT, the higher current conditions increase the magnetic permeability of the core, which operates more efficiently at a flux density corresponding to 25% rather than 12.5% of the rated current. As a result, the current error of the corrected iCT becomes excessively positive.

The wideband characteristics of the tested RCs and iCTs for the 1st–13th harmonics, presented in Figure 8, Figure 9, Figure 10, Figure 11, Figure 12 and Figure 13, show that the current-to-voltage conversion errors (RCs), current errors (iCTs), additional phase shift (RCs), and phase displacement (iCTs) do not exceed the limit values of ±5% and ±5° for the WB0 accuracy class of IEC 61869-1 [28]. While the tested RCs exhibit wideband performance comparable to that of the iCTs, they may experience current-to-voltage conversion errors of approximately 1% at 50 Hz depending on the primary conductor position, ambient temperature, and other external factors. These values in the least demanding case at lowest current exceed the limits defined in IEC 61869-2/10, which are 0.75% for accuracy classes 0.2 [27,29]. However, in the case when accuracy class 0.5 is declared, these values in the least demanding case at lowest current do not exceed the limits defined in IEC 61869-2/10, which are 1.5% for accuracy classes 0.5. In the case of the additional phase shift at higher frequency, only one coil RCd70 shows similar performance with iCTs. The TiCT_3 failed to meet the most restrictive (at highest current) IEC 61869-2 requirements for accuracy class 0.2, as its secondary winding load was below the minimum 1.25 VA. However, the other two TiCTs successfully met the declared accuracy class requirements at 50 Hz (Table 2).

**Figure 14:**
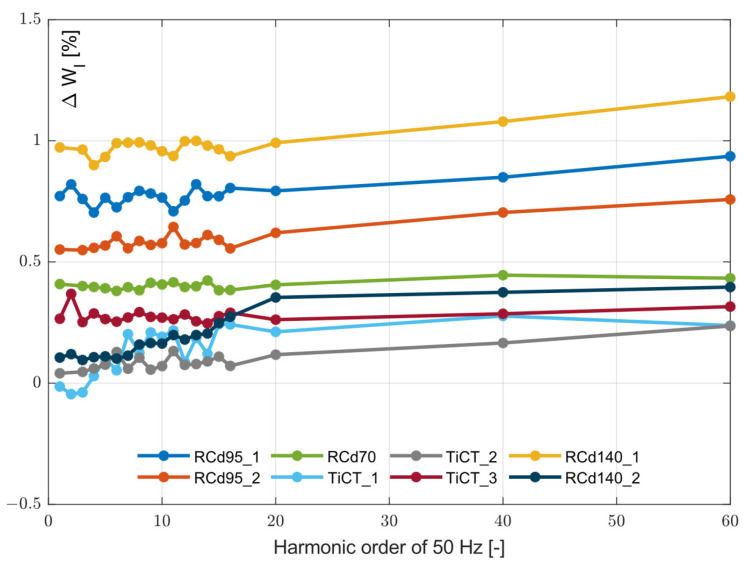
Current-to-voltage conversion (RCs) and current (iCTs) errors for the 1st–60th harmonics, relative to the RiCT at 50 A (WB1 compliance evaluation).

**Figure 15:**
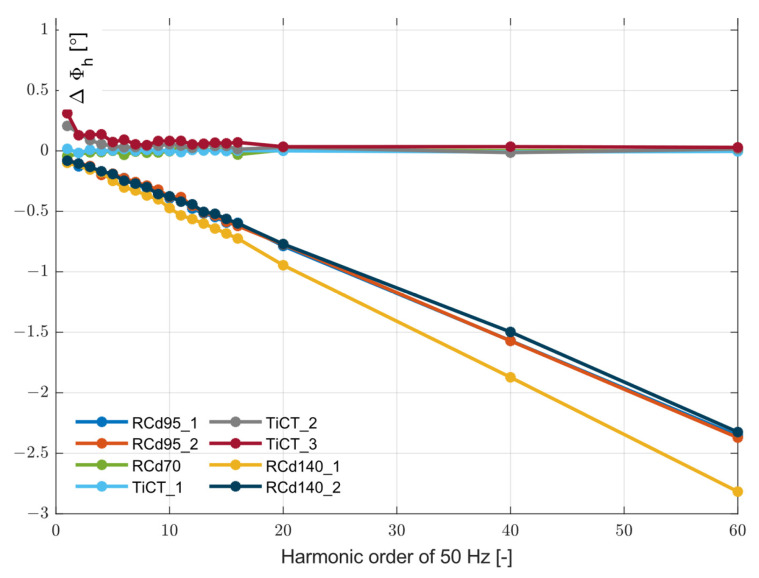
Additional phase shift (RCs) and phase displacement (iCTs) for the 1st–60th harmonics, relative to the RiCT at 50 A (WB1 compliance evaluation).

**Figure 16:**
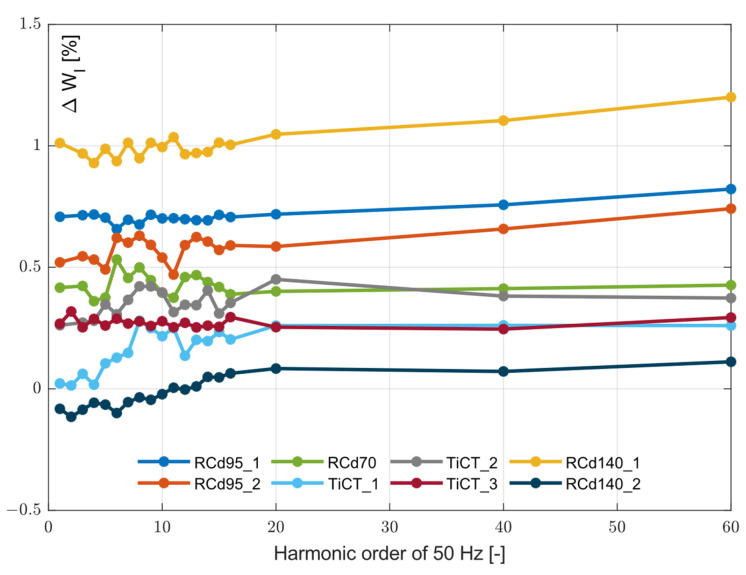
Current-to-voltage conversion (RCs) and current (iCTs) errors for the 1st–60th harmonics, relative to the RiCT at 100 A (WB1 compliance evaluation).

**Figure 17:**
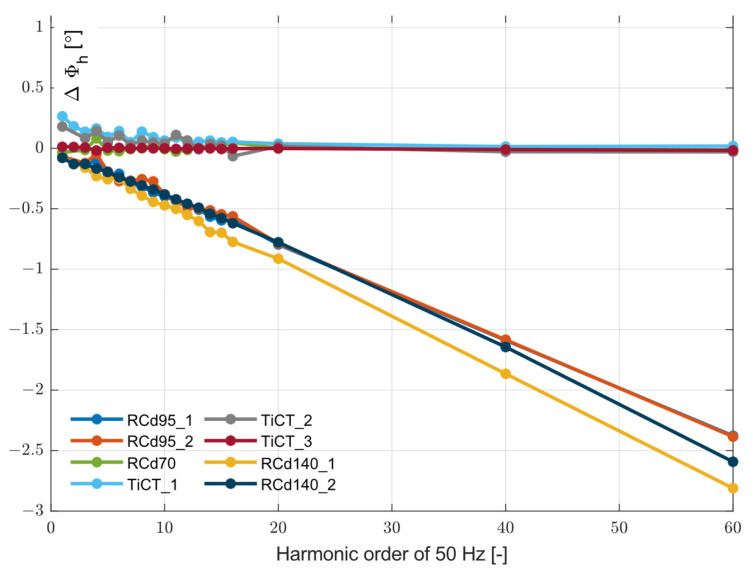
Additional phase shift (RCs) and phase displacement (iCTs) for the 1st–60th harmonics, relative to the RiCT at 100 A (WB1 compliance evaluation).

**Figure 18:**
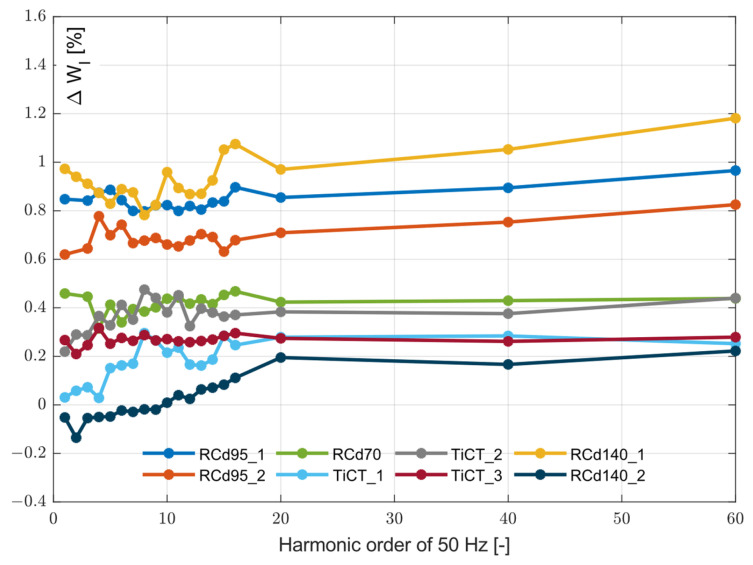
Current-to-voltage conversion (RCs) and current (iCTs) errors for the 1st–60th harmonics, relative to the RiCT at 150 A (WB1 compliance evaluation).

**Figure 19:**
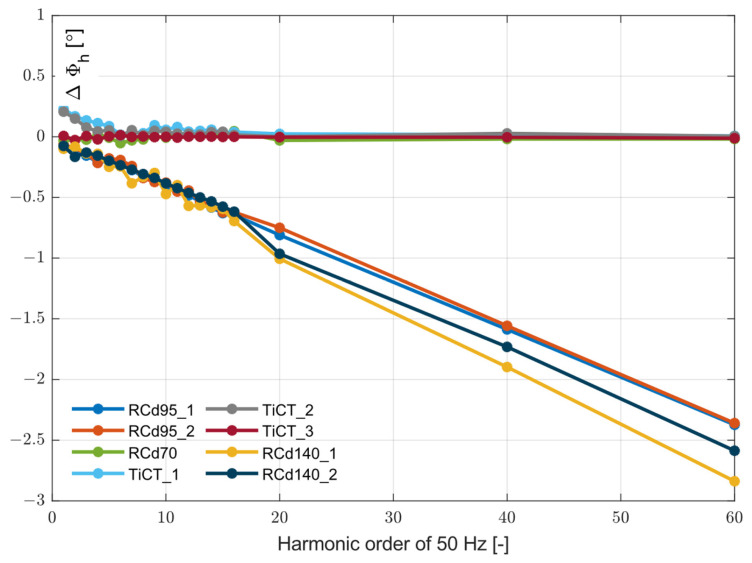
Additional phase shift (RCs) and phase displacement (iCTs) for the 1st–60th harmonics, relative to the RiCT at 150 A (WB1 compliance evaluation).

The wideband characteristics of the tested RCs and iCTs for the 1st–60th harmonics are illustrated in Figure 14, Figure 15, Figure 16, Figure 17, Figure 18 and Figure 19. These figures again compare current-to-voltage conversion errors and current errors as well as additional phase shift introduced by tested RCs and phase displacement of iCTs determined relative to the RiCT at three primary current levels: 50 A, 100 A, and 150 A. The results are further analyzed for compliance with requirements of the IEC 61869-1 WB1 accuracy class.

In the extended frequency range up to 3 kHz, it can be seen that the RCd140_2 current-to-voltage conversion error increases with frequency, as it has the highest sensitivity of 500 mV/1 kA, resulting in the highest parasitic capacitance and leakage inductance. However, the additional phase shift is similar to that of the other tested RCs with 100 mV/1 kA sensitivity. In both respects, RCd70 shows the best linear performance, as its sensitivity is only 22.5 mV/1 kA, which results in reduced parasitic capacitance and leakage inductance. TiCT_1 also exhibits interesting behavior, as its current error increases from approximately 0% to 0.3% and then stabilizes. This is due to the fact that as the frequency increases, the excitation current of its magnetic core is reduced because the mutual reactance of the iCT’s windings increases (despite some obvious decrease in magnetic permeability, albeit to a lesser extent), causing a reduction in the magnetization current, along with a reduced magnetic flux density in the magnetic core. As a result of the increased frequency, also the active power losses are reduced without any change in the secondary winding load, and therefore the current associated with active power losses is also reduced. In the case of TiCT_1, the phase displacement also decreases with frequency for the same reason.

Increasing the current of all iCTs from 50 A to 100 A (or even 150 A in the next case) does not cause any significant change to their behavior with frequency, similar to the case of RCs, but in a limited range due to the presence of a magnetic core and its possible saturation. This was obtained due to the maximum reduction of their secondary winding load, as it results only from the current sensing resistor of the DPM module. This reduces the magnetic flux density, but also limits its increase caused by the increase of the primary current value. Therefore, it should be noted that deterioration of the secondary winding load in the case of iCTs helps to obtain a more linear current error and phase displacement with change of frequency. However, in the case of current error, a too high positive value may appear. Therefore, for best performance, iCTs should be designed so that no secondary winding turns correction is required.

Presented in Figure 14, Figure 15, Figure 16, Figure 17, Figure 18 and Figure 19, frequency characteristics of tested RCs and iCTs for the 1st–60th harmonics show that the current-to-voltage conversion errors (RCs), current errors (iCTs), additional phase shift (RCs), and phase displacement (iCTs) do not exceed the limit values defined in the standard IEC 61869-1 for the wideband accuracy class 0.1-WB1 starting from the 2nd harmonic: maximum current error/phase displacement of ±1%/±1° (≤1 kHz/20th harmonic), ±2%/±2° (≤1.5 kHz/30th harmonic), and ±5%/±5° (≤3 kHz/60th harmonic). However, it should be noted that the most restrictive requirements (at highest current: 0.2%/0.17°) of 0.2 accuracy class defined by the IEC 61869-2 for 50 Hz, despite the declaration by the manufacturer of 100 mV\1 kA RCs (Table 1), are not met. Therefore, the combined accuracy class is 0.5-WB1 in the case of almost all RCs and all tested iCTs. Only in the case of the RCd140_2 is the compliance with the 0.2-WB1 confirmed. Moreover, in the case of the additional phase shift at higher frequency, the coils RCd140_1(2) and RCd95_1(2) show significantly higher additional phase shift than tested iCTs and RCd70, reaching about −2.5°.

## 5. Discussion

The tests demonstrated that conventional instrument transformers (Figure 1) with a ferromagnetic core can be successfully utilized for the measurement of distorted currents. However, it should be noted that their transformation accuracy is inherently dependent on the primary current magnitude and the secondary winding burden [1,3,7]. Therefore, to improve their wideband performance, the tests were conducted with the secondary winding burden limited solely to the current-sensing resistor of the digital power meter. As a result, the TiCT_3 failed to meet its manufacturer-declared accuracy class of 0.2 according to IEC 61869-2. In iCTs where secondary winding turns correction is applied, an unacceptably reduced burden (specifically below 25% of the rated burden or smallest permissible 1 VA) causes the current error to become excessively positive, thereby exceeding the limiting values for accuracy class 0.2. Moreover, such iCTs will not maintain a linear current error characteristic with respect to frequency—as it increases, the excitation current decreases due to the higher mutual reactance between the windings and the reduced magnetic flux density in the magnetic core [1,3]. Consequently, while reaching required accuracy class at increased secondary winding load, the current error will shift in the positive direction at higher frequencies. This trend will continue until the decline in magnetic permeability exceeds the frequency-dependent increase in mutual reactance. Furthermore, the self-generation phenomenon can be observed in iCTs, manifesting as a rapid increase in current error and phase displacement at low-order harmonics (specifically the 3rd and 5th). Due to the non-linear magnetization characteristics of the magnetic core, these harmonics arise in the secondary current. Depending on the phase angle of the transformed low-order harmonics relative to the fundamental component of the distorted primary current, they superimpose at different angles, thereby altering the transformation errors of the iCT. This phenomenon intensifies with an increased secondary winding burden and a higher primary current, thereby degrading the wideband performance [1,3].

Rogowski coils (RCs) proved to be a viable alternative to inductive current transformers (iCTs) due to their insensitivity to the primary current value; nevertheless, most of the tested designs exhibited a significant increase in the introduced additional phase shift as the harmonic order increased. Among the tested RCs, the smallest additional phase shift at higher frequencies was observed in the unit (RCd70) with the lowest sensitivity (22.5 mV/1 kA) and, simultaneously, the smallest coil diameter (70 mm). This is attributed to the reduction in internal parasitic capacitance and leakage inductance (Figure 2). However, because this current sensor yields the lowest output voltage for a given primary current compared to the other evaluated RCs, it imposes stricter requirements on the sensitivity of the measurement device. The low-level output signal necessitates high-resolution data acquisition to prevent quantization errors and makes the system more susceptible to external noise. It meets the least restrictive 0.2 accuracy class requirements of IEC 61869-2/10 in the entire tested frequency range and its most restrictive accuracy requirements of class 0.5 in the entire tested frequency range. This analyzed aspect concerns evaluation of the same requirements as defined by the standards but in the frequency range extended for higher harmonics. The least restrictive criteria refer to the fact that different limiting values are defined for various primary current levels in iCTs due to the nonlinear magnetization characteristics of the magnetic core. The widest error margins are specified for the lowest primary currents relative to the rated value, since the excitation current under these conditions becomes proportionally larger compared to the primary current. One RC with a manufacturer-declared accuracy class of 0.2 according to IEC 61869-10/2 at 50 Hz (RCd140_1) exceeded even the least restrictive limiting value ±0.75% for current-to-voltage conversion error. This deviation was probable caused by an additional error given by its large diameter (140 mm) relative to the primary conductor diameter (15 mm) required for a highest RMS current of 200 A. Adjusting the cable position failed to reduce the current-to-voltage conversion error. Conversely, when analyzing accuracy class 0.5 under the most stringent requirements (±0.5%/°), the two RCs (RCd140_2 and RCd70) met the IEC 61869-2/10 standards for a 50 Hz component, as declared by their manufacturers. It should be noted that the conversion accuracy of the RCd140_2 (140 mm diameter) is highly dependent on the conductor position. The low current-to-voltage conversion error was obtained in a strictly central position, but it can increase to 1% depending on the primary cable’s position within the coil.

In Table 3 the summary of the results for all tested current transducers is presented.

The results reveled that the requirements of the 0.5-WB1 class are satisfied in the frequency range up to the 60th harmonic by all tested transducers, while in the case of the tested RC labelled RCd140_2, the more restrictive 0.2-WB1 class is also satisfied. However, when considering solely the limiting values of current error and phase displacement from the 2nd harmonic onwards, the requirements of the 0.1-WB1 class are fulfilled. The findings confirmed that the reference iCT exhibited the highest accuracy, outperforming the electronic current transformer (eCT). It must be emphasized that such wideband performance necessitates an appropriate magnetic core capable of ensuring the required transformation accuracy without compensating the secondary winding turns [1]. The proposed additional analysis of the IEC 61869-2/10 accuracy class requirements, extended from the required 50/60 Hz to the wideband frequency range to apply more stringent criteria, reveals significant differences in the high-frequency performance of the tested transducers. The reference inductive current transformer (RiCT) and zero-flux electronic current transformer (eCT) demonstrated superior transformation accuracy, satisfying the most restrictive requirements of class 0.1 across the entire tested frequency range. Among the evaluated Rogowski coils, the RCd70 unit exhibited the best wideband conversion characteristics, fulfilling the most restrictive limits of class 0.5 and the least restrictive limits of class 0.2 over the entire frequency spectrum. The RCd140_2 and RCd95_1 coils showed moderate wideband conversion capabilities, meeting the requirements of class 0.2 up to the 10th harmonic and class 0.5 up to the 40th harmonic, under the most and least restrictive criteria, respectively. Conversely, the RCd140_1 unit only maintained the least restrictive conversion limits of class 0.5 up to the 30th harmonic. Finally, all conventional iCTs met the requirements of class 0.5 over the entire tested range.

## 6. Conclusions

The evaluation of low-voltage instrument current transformers and current sensors under the extended wideband frequency range of primary current harmonics reveals similar wideband performance up to 3 kHz (60th higher harmonic). The proposed additional analysis of the IEC 61869-2/10 accuracy class requirements, extended from the required 50/60 Hz to the wideband frequency range to apply more stringent criteria, reveals some differences in the high-frequency performance of the tested transducers. Both the reference inductive current transformer and the electronic current transformer demonstrated the highest wideband performance, maintaining compliance with the most restrictive 0.1 accuracy class across the entire tested frequency range. Among tested Rogowski coils, one exhibited excellent characteristics, satisfying both the least restrictive 0.2 class and the most restrictive 0.5 class criteria over the complete evaluated frequency range. Conversely, the wide-frequency accuracy of the remaining coils was more limited. For instance, one coil met the least restrictive 0.5 class requirements only up to the 30th harmonic. Two other coils maintained 0.2 class compliance up to the 10th harmonic and 0.5 class compliance up to the 40th harmonic, evaluated under their respective most and least restrictive criteria.

The tested inductive current transformer ensured continuous compliance with the 0.5 class across the entire tested frequency range, successfully accommodating the different limiting values defined for 5%, 20%, 100%, and 120% of the rated primary current. The experimental results clearly demonstrate that they are able to successfully maintain the 0.5-WB1 (and WB0) accuracy class defined in the standard IEC 61869-1 across the entire tested frequency range of higher harmonics. In the case of inductive current transformers, the reduction of secondary burden is advised in order to obtain more linear frequency response. However, this may cause increased current error at low frequency. Tested Rogowski coils exhibit various current-to-voltage conversion accuracy and introduced phase shift. It was shown that a manufacturer’s declaration of a 0.2 accuracy class at the nominal 50/60 Hz frequency does not automatically ensure such performance under specific geometric conditions, for instance when the primary conductor diameter is disproportionately small relative to the coil’s aperture. Therefore, they actual wideband behavior is heavily dictated by their specific physical design. The Rogowski coils with smaller diameters and lower sensitivities exhibit the most linear wideband characteristics concerning the introduced additional phase shift of output voltage harmonics in relation to the primary current harmonic of the same order. Tested Rogowski coils are typically able to ensure 0.5-WB1 compliance across the entire evaluated frequency range up to 3 kHz. It has also been shown that 0.2-WB1 accuracy class compliance is achievable by certain units in a conventional configuration.

## Figures and Tables

**Table 1:** Assigned percentage values of higher harmonics relative to the fundamental current.

Harmonic Order	3	5, 10	2, 4, 6, 7, 8, 12, 14, 16, 20, 40	9, 11, 13, 15, 60, 80, 100, 120, 140, 160, 180, 200
Percentage of 1st harmonic	15%	5%	2%	1%

**Table 2:** List of used abbreviations for the transducers and their main technical specifications.

Device Designation	Sensitivity [mV/1 kA] or Current Ratio [A/A]	Accuracy Class IEC 61869-2/10 (50 Hz) or Maximum Current Error [%] and Phase Displacement [°]
RiCT	250/5	±0.02%/°
eCT	200/0.2	10 Hz–5 kHz: ±0.1%/°; 5 kHz–100 kHz: ±2.0%/0.5°
RCd95_1	100 (same RC two units)	0.2
RCd95_2		
RCd140_1	100	
RCd140_2	500	0.5
RCd70	22.5	
TiCT_1	250/1	0.5
TiCT_2	400/1	0.5S
TiCT_3	100/1	0.2

**Table 3:** Summary of the results for all tested current transducers.

Tested RC/iCT	Compliance with the Requirements of IEC 61869-2/10 Class for 50 Hz Sinusoidal Current in Extended Frequency Range	WB Accuracy Class Defined in the Standard IEC 61869-1	Compliance with the WB Class Requirements from 2nd Harmonic
RiCT	0.1 (most restrictive): entire tested range	WB0 and 0.1-WB1	0.1-WB1
eCT			
RCd140_1	0.5 (least restrictive): up to the 30th harmonic	WB0 and 0.5-WB1	
RCd140_2	0.2 (most restrictive): up to the 10th harmonic 0.5 (most restrictive): up to the 40th harmonic	WB0 and 0.2-WB1	
RCd95_1	0.2 (least restrictive): up to the 10th harmonic 0.5 (least restrictive): up to the 40th harmonic	WB0 and 0.5-WB1	
RCd95_2			
RCd70	0.2 (least restrictive): entire tested range 0.5 (most restrictive): entire tested range		
TiCT_1	0.5 (taking into consideration different limiting values as defined for 5%, 20%, 100% and 120% of rated primary current): entire tested range		
TiCT_2			
TiCT_3			

## Data Availability

Data is provided within the manuscript.
